# Body Mass Index and Risk for COVID-19–Related Hospitalization, Intensive Care Unit Admission, Invasive Mechanical Ventilation, and Death — United States, March–December 2020

**DOI:** 10.15585/mmwr.mm7010e4

**Published:** 2021-03-12

**Authors:** Lyudmyla Kompaniyets, Alyson B. Goodman, Brook Belay, David S. Freedman, Marissa S. Sucosky, Samantha J. Lange, Adi V. Gundlapalli, Tegan K. Boehmer, Heidi M. Blanck

**Affiliations:** ^1^Division of Nutrition, Physical Activity, and Obesity, National Center for Chronic Disease Prevention and Health Promotion, CDC; ^2^CDC COVID-19 Response Team, CDC.

Obesity* is a recognized risk factor for severe COVID-19 (*1*,*2*), possibly related to chronic inflammation that disrupts immune and thrombogenic responses to pathogens (*3*) as well as to impaired lung function from excess weight (*4*). Obesity is a common metabolic disease, affecting 42.4% of U.S. adults (*5*), and is a risk factor for other chronic diseases, including type 2 diabetes, heart disease, and some cancers.^†^ The Advisory Committee on Immunization Practices considers obesity to be a high-risk medical condition for COVID-19 vaccine prioritization (*6*). Using data from the Premier Healthcare Database Special COVID-19 Release (PHD-SR),^§^ CDC assessed the association between body mass index (BMI) and risk for severe COVID-19 outcomes (i.e., hospitalization, intensive care unit [ICU] or stepdown unit admission, invasive mechanical ventilation, and death). Among 148,494 adults who received a COVID-19 diagnosis during an emergency department (ED) or inpatient visit at 238 U.S. hospitals during March–December 2020, 28.3% had overweight and 50.8% had obesity. Overweight and obesity were risk factors for invasive mechanical ventilation, and obesity was a risk factor for hospitalization and death, particularly among adults aged <65 years. Risks for hospitalization, ICU admission, and death were lowest among patients with BMIs of 24.2 kg/m^2^, 25.9 kg/m^2^, and 23.7 kg/m^2^, respectively, and then increased sharply with higher BMIs. Risk for invasive mechanical ventilation increased over the full range of BMIs, from 15 kg/m^2^ to 60 kg/m^2^. As clinicians develop care plans for COVID-19 patients, they should consider the risk for severe outcomes in patients with higher BMIs, especially for those with severe obesity. These findings highlight the clinical and public health implications of higher BMIs, including the need for intensive COVID-19 illness management as obesity severity increases, promotion of COVID-19 prevention strategies including continued vaccine prioritization (*6*) and masking, and policies to ensure community access to nutrition and physical activities that promote and support a healthy BMI.

Data for this study were obtained from PHD-SR, a large, hospital-based, all-payer database. Among the approximately 800 geographically dispersed U.S. hospitals that reported both inpatient and ED data to this database, 238 reported patient height and weight information and were selected for this study. The sample included patients aged ≥18 years with measured height and weight and an ED or inpatient encounter with an *International Classification of Diseases, Tenth Revision, Clinical Modification* (ICD-10-CM) code of U07.1 (COVID-19, virus identified) during April 1–December 31, 2020, or B97.29 (other coronavirus as the cause of diseases classified elsewhere; recommended before April 2020 release of U07.1) during March 1–April 30, 2020.^¶^ BMI was calculated using heights and weights measured during the health care encounter closest to the patient’s ED or hospital encounter for COVID-19 in the database.** BMI was classified into the following categories: underweight (<18.5 kg/m^2^), healthy weight (18.5–24.9 kg/m^2^ [reference]), overweight (25–29.9 kg/m^2^), and obesity (four categories: 30–34.9 kg/m^2^, 35–39.9 kg/m^2^, 40–44.9 kg/m^2^, and ≥45 kg/m^2^).

Frequencies and percentages were used to describe the patient sample. Multivariable logit models were used to estimate adjusted risk ratios (aRRs) between BMI categories and four outcomes of interest: hospitalization (reference = ED patients not hospitalized) and ICU admission, invasive mechanical ventilation, and death among hospitalized patients (reference = hospitalized patients without the outcome and who did not die).^††^ Analyses were then stratified by age (<65 years versus ≥65 years). Multivariable logit models were used to estimate risks for the outcomes of interest based on continuous BMI (modeled as fractional polynomials to account for nonlinear associations) (*7*).^§§^ Risks were reestimated for different age categories, after including interactions between age category and BMI.

Models used robust standard errors clustered on hospital identification and included age,^¶¶^ sex, race/ethnicity, payer type, hospital urbanicity, hospital U.S. Census region, and admission month as control variables. Models did not adjust for other underlying medical conditions known to be risk factors for COVID-19,*** because most of these conditions represent intermediate variables on a causal pathway from exposure (i.e., BMI) to outcome. A sensitivity analysis adjusting for these conditions was performed.^†††^ A second sensitivity analysis used multiple imputation for missing BMIs. Analyses were conducted using R software (version 4.0.3; The R Foundation) and Stata (version 15.1, StataCorp). This activity was reviewed by CDC and conducted consistent with applicable federal law and CDC policy.^§§§^

Among 3,242,649 patients aged ≥18 years with documented height and weight who received ED or inpatient care in 2020, a total of 148,494 (4.6%) had ICD-10-CM codes indicating a diagnosis of COVID-19 (Table). Among 71,491 patients hospitalized with COVID-19 (48.1% of all COVID-19 patients), 34,896 (48.8%) required ICU admission, 9,525 (13.3%) required invasive mechanical ventilation, and 8,348 (11.7%) died. Approximately 1.8% of patients had underweight, 28.3% had overweight, and 50.8% had obesity. Compared with the total PHD-SR cohort, patients with COVID-19–associated illness were older (median age of 55 years versus 49 years) and had a higher crude prevalence of obesity (50.8% versus 43.1%).

**TABLE:** Characteristics of patients aged ≥18 years with a COVID-19–related emergency department or inpatient hospital visit — Premier Healthcare Database Special COVID-19 Release (PHD-SR),* United States, March–December 2020

Characteristic^†^	No. (%)^§^					
	Total cohort in database	Patients with COVID-19				
		Total cohort	Hospitalized	Hospitalized, ICU care	Hospitalized, IMV	Hospitalized, died
**Total**	**3,242,649 (100.0)**	**148,494 (100.0)**	**71,491 (100.0)**	**34,896 (100.0)**	**9,525 (100.0)**	**8,348 (100.0)**
**Sex**						
Female	**1,852,609 (57.1)**	79,624 (53.6)	35,253 (49.3)	15,601 (44.7)	3,818 (40.1)	3,468 (41.5)
Male	**1,390,040 (42.9)**	68,870 (46.4)	36,238 (50.7)	19,295 (55.3)	5,707 (59.9)	4,880 (58.5)
**Age, yrs, median (IQR)**	**49 (32–66)**	55 (38–70)	65 (52–77)	66 (54–77)	67 (57–76)	74 (65–83)
**Age group, yrs**						
18–39	**1,230,684 (38.0)**	39,545 (26.6)	8,979 (12.6)	2,907 (8.3)	525 (5.5)	126 (1.5)
40–49	**431,355 (13.3)**	20,638 (13.9)	6,869 (9.6)	3,258 (9.3)	761 (8.0)	277 (3.3)
50–64	**703,229 (21.7)**	37,877 (25.5)	19,059 (26.7)	9,784 (28.0)	2,855 (30.0)	1,555 (18.6)
65–74	**422,407 (13.0)**	23,158 (15.6)	15,406 (21.5)	8,291 (23.8)	2,683 (28.2)	2,221 (26.6)
≥75	**454,974 (14.0)**	27,276 (18.4)	21,178 (29.6)	10,656 (30.5)	2,701 (28.4)	4,169 (49.9)
**Race/Ethnicity**						
Hispanic or Latino	**337,234 (10.4)**	29,576 (19.9)	12,303 (17.2)	6,197 (17.8)	1,619 (17.0)	1,244 (14.9)
White, non-Hispanic	**2,064,343 (63.7)**	75,659 (51.0)	40,292 (56.4)	19,413 (55.6)	5,256 (55.2)	5,167 (61.9)
Black, non-Hispanic	**597,909 (18.4)**	30,306 (20.4)	12,735 (17.8)	6,377 (18.3)	1,697 (17.8)	1,261 (15.1)
Asian, non-Hispanic	**67,286 (2.1)**	3,536 (2.4)	1,662 (2.3)	668 (1.9)	231 (2.4)	159 (1.9)
Other	**130,723 (4.0)**	6,729 (4.5)	3,252 (4.5)	1,619 (4.6)	516 (5.4)	353 (4.2)
Unknown	**45,154 (1.4)**	2,688 (1.8)	1,247 (1.7)	622 (1.8)	206 (2.2)	164 (2.0)
**Payer type**						
Commercial	**1,002,345 (30.9)**	49,366 (33.2)	17,543 (24.5)	8,130 (23.3)	1,935 (20.3)	887 (10.6)
Medicare	**997,984 (30.8)**	55,598 (37.4)	38,598 (54.0)	19,901 (57.0)	5,661 (59.4)	6,380 (76.4)
Medicaid	**640,338 (19.7)**	22,213 (15.0)	8,358 (11.7)	3,278 (9.4)	1,021 (10.7)	540 (6.5)
Charity/Indigent/Self-Pay	**416,485 (12.8)**	7,179 (4.8)	2,246 (3.1)	1,086 (3.1)	254 (2.7)	130 (1.6)
Other/Unknown	**185,497 (5.7)**	14,138 (9.5)	4,746 (6.6)	2,501 (7.2)	654 (6.9)	411 (4.9)
**Body mass index (kg/m^2^)**						
<18.5 (underweight)	**79,988 (2.5)**	2,674 (1.8)	1,730 (2.4)	865 (2.5)	169 (1.8)	273 (3.3)
18.5–24.9 (healthy weight)	**829,474 (25.6)**	28,349 (19.1)	14,111 (19.7)	6,891 (19.7)	1,550 (16.3)	1,957 (23.4)
25–29.9 (overweight)	**936,132 (28.9)**	41,973 (28.3)	19,847 (27.8)	9,661 (27.7)	2,435 (25.6)	2,277 (27.3)
≥30 (obesity)	**1,397,055 (43.1)**	75,498 (50.8)	35,803 (50.2)	17,479 (50.1)	5,371 (56.3)	3,841 (46.0)
30–34.9	**674,575 (20.8)**	34,608 (23.3)	16,338 (22.9)	7,883 (22.6)	2,300 (24.1)	1,830 (21.9)
35–39.9	**373,226 (11.5)**	20,262 (13.6)	9,476 (13.3)	4,601 (13.2)	1,399 (14.7)	960 (11.5)
40–44.9 (severe obesity)	**187,046 (5.8)**	10,739 (7.2)	5,015 (7.0)	2,438 (7.0)	783 (8.2)	517 (6.2)
≥45 (severe obesity)	**162,208 (5.0)**	9,889 (6.7)	4,974 (7.0)	2,557 (7.3)	889 (9.3)	534 (6.4)
**Hospital U.S. Census region^¶^**						
Midwest	**683,575 (21.1)**	33,800 (22.8)	16,305 (22.8)	6,907 (19.8)	2,279 (23.9)	1,795 (21.5)
Northeast	**476,367 (14.7)**	18,276 (12.3)	10,758 (15.0)	3,641 (10.4)	1,557 (16.3)	1,639 (19.6)
South	**1,988,506 (61.3)**	94,555 (63.7)	43,616 (61.0)	23,955 (68.6)	5,567 (58.4)	4,812 (57.6)
West	**94,201 (2.9)**	1,863 (1.3)	812 (1.1)	393 (1.1)	122 (1.3)	102 (1.2)

**Abbreviations:** ICU = intensive care or stepdown unit; IMV = invasive mechanical ventilation; IQR = interquartile range.

* Data in PHD-SR, formerly known as the PHD COVID-19 Database, are released every 2 weeks; release date March 2, 2021, access date March 3, 2021. http://offers.premierinc.com/rs/381-NBB-525/images/PHD_COVID-19_White_Paper.pdf

^†^ Categories might not sum to 100% because of rounding or because they are not mutually exclusive.

^§^ Columns are not mutually exclusive.

^¶^ *Northeast*: Connecticut, Maine, Massachusetts, New Hampshire, New Jersey, New York, Pennsylvania, Rhode Island, Vermont; *Midwest*: Illinois, Indiana, Iowa, Kansas, Michigan, Minnesota, Missouri, Nebraska, North Dakota, Ohio, South Dakota, Wisconsin; *South*: Alabama, Arkansas, Delaware, District of Columbia, Florida, Georgia, Kentucky, Louisiana, Maryland, Mississippi, North Carolina, Oklahoma, South Carolina, Tennessee, Texas, Virginia, West Virginia; *West*: Alaska, Arizona, California, Colorado, Hawaii, Idaho, Montana, Nevada, New Mexico, Oregon, Utah, Washington, and Wyoming.

Obesity was a risk factor for both hospitalization and death, exhibiting a dose-response relationship with increasing BMI category: aRRs for hospitalization ranged from 1.07 (95% confidence interval [CI = 1.05–1.09]) for patients with a BMI of 30–34.9 kg/m^2^ to 1.33 (95% CI = 1.30–1.37) for patients with a BMI ≥45 kg/m^2^ (Figure 1) compared with those with a BMI of 18.5–24.9 kg/m^2^ (healthy weight); aRRs for death ranged from 1.08 (95% CI = 1.02–1.14) for those with a BMI of 30–34.9 kg/m^2^ to 1.61 (95% CI = 1.47–1.76) for those with a BMI ≥45 kg/m^2^. Severe obesity was associated with ICU admission, with aRRs of 1.06 (95% CI = 1.03–1.10) for patients with a BMI of 40–44.9 kg/m^2^ and 1.16 (95% CI = 1.11–1.20) for those with a BMI ≥45 kg/m^2^. Overweight and obesity were risk factors for invasive mechanical ventilation, with aRRs ranging from 1.12 (95% CI = 1.05–1.19) for a BMI of 25–29.9 kg/m^2^ to 2.08 (95% CI = 1.89–2.29) for a BMI ≥45 kg/m^2^. Associations with risk for hospitalization and death were pronounced among adults aged <65 years: aRRs for patients in the highest BMI category (≥45 kg/m^2^) compared with patients with healthy weights were 1.59 (95% CI = 1.52–1.67) for hospitalization and 2.01 (95% CI = 1.72–2.35) for death.

**FIGURE 1:**
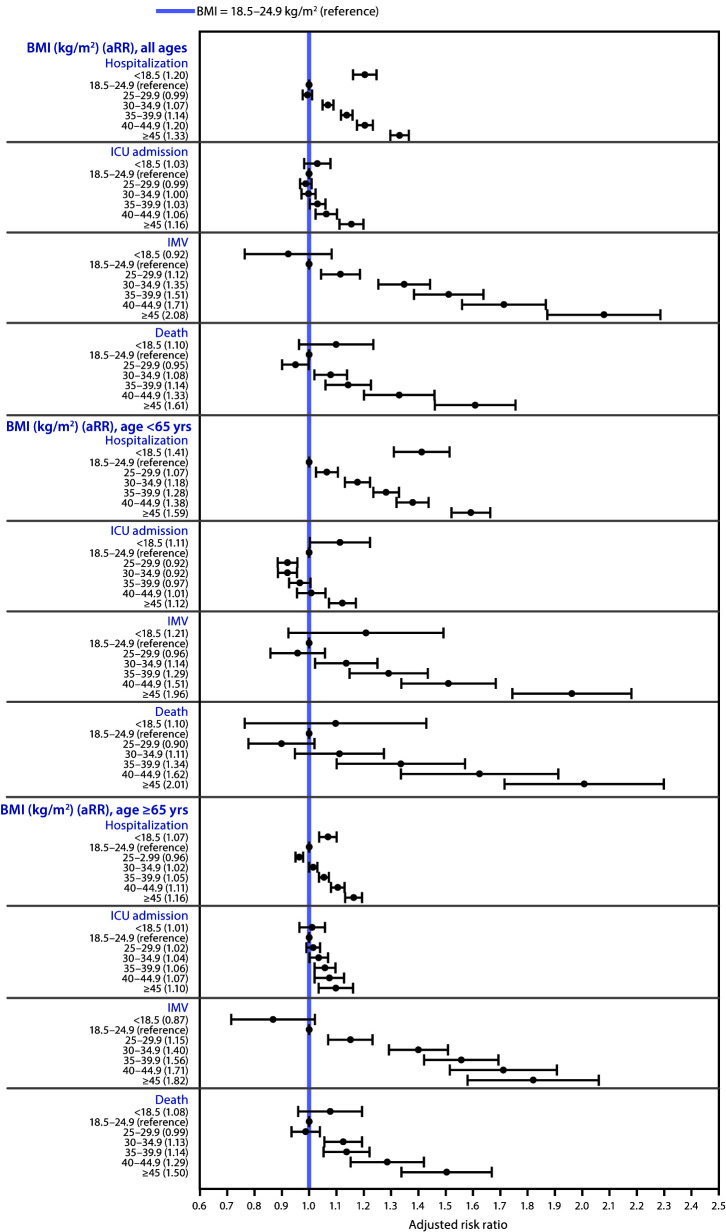
Association between body mass index (BMI) and severe COVID-19–associated illness* among adults aged ≥18 years, by age group — Premier Healthcare Special COVID-19 Release (PHD-SR),^†^ United States, March–December 2020^§^ **Abbreviations:** aRR = adjusted risk ratio; ICU = intensive care or stepdown unit; IMV = invasive mechanical ventilation. * Illness requiring hospitalization, ICU admission, or IMV or resulting in death. ^†^ Data in PHD-SR, formerly known as the PHD COVID-19 Database, are released every 2 weeks; release date March 2, 2021, access date March 3, 2021. http://offers.premierinc.com/rs/381-NBB-525/images/PHD_COVID-19_White_Paper.pdf ^§^ Each panel contains the results of a single logit model, adjusted for BMI category, age, sex, race/ethnicity, payer type, hospital urbanicity, hospital U.S. Census region, and admission month as control variables. Age group (18–39 [reference], 40–49, 50–64, 65–74, and ≥75 yrs) was used as a control variable in the models that included patients of all ages (first four panels), whereas continuous age as cubic polynomial was used as a control variable in models stratified by age (<65 and ≥65 yrs). Risk for hospitalization was estimated in the full sample; risk for ICU admission, IMV, and death were estimated in the hospitalized sample. Patients who died without requiring ICU admission or IMV were excluded from the sample when estimating the model with outcome of ICU admission or IMV, respectively.

Patients with COVID-19 with underweight had a 20% (95% CI = 16%–25%) higher risk for hospitalization than did those with a healthy weight. Patients aged <65 years with underweight were 41% (95% CI = 31%–52%) more likely to be hospitalized than were those with a healthy weight, and patients aged ≥65 years with underweight were 7% (95% CI = 4%–10%) more likely to be hospitalized.

A J-shaped (nonlinear) relationship was observed between continuous BMI and risk for three outcomes. Risk for hospitalization, ICU admission, and death were lowest at BMIs of 24.2 kg/m^2^, 25.9 kg/m^2^, and 23.7 kg/m^2^, respectively, and then increased sharply with higher BMIs (Figure 2). Estimated risk for invasive mechanical ventilation increased over the full range of BMIs, from 15 kg/m^2^ to 60 kg/m^2^. Estimated risks for hospitalization and death were consistently higher for older age groups; however, within each age group, risk increased with higher BMIs.

**FIGURE 2:**
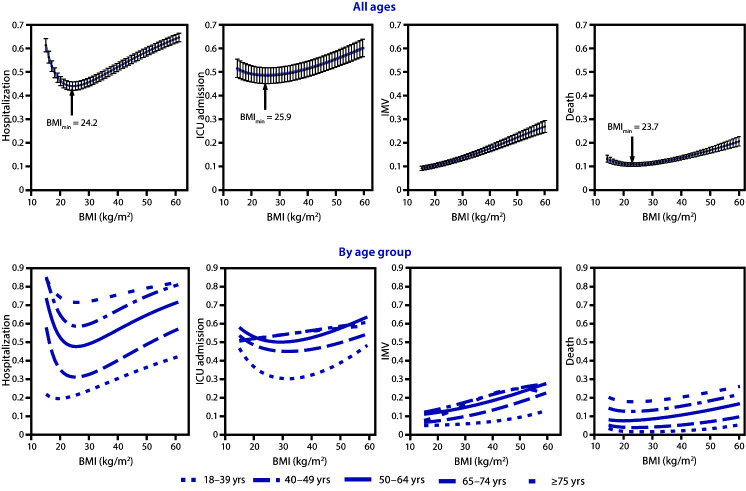
Estimated risk for severe COVID-19–associated illness* among adults aged ≥18 years, by body mass index (BMI) and age group — Premier Healthcare Special COVID-19 Release (PHD-SR),^†^ United States, March–December, 2020^§^ **Abbreviations:** ICU = intensive care or stepdown unit; IMV = invasive mechanical ventilation. * Illness requiring hospitalization, ICU admission, or IMV or resulting in death. ^†^ Data in PHD-SR, formerly known as the PHD COVID-19 Database, are released every 2 weeks; release date March 2, 2021, access date March 3, 2021. http://offers.premierinc.com/rs/381-NBB-525/images/PHD_ COVID-19_White_Paper.pdf ^§^ Each panel contains the results of a single logit model, adjusted for BMI (as fractional polynomials), age group (18–39 [reference], 40–49, 50–64, 65–74, and ≥75 yrs), sex, race/ethnicity, payer type, hospital urbanicity, hospital U.S. Census region, and admission month as control variables. Confidence intervals are shown by error bars. The bottom panels also include interactions between BMI (as fractional polynomials) and age group. Risk for hospitalization was estimated in the full sample; risk for ICU admission, IMV, and death were estimated in the hospitalized sample. Patients who died without requiring ICU admission or IMV were excluded from the sample when estimating the model with outcome of ICU admission or IMV, respectively. The best fitting models included the following fractional polynomials of BMI: BMI^-2^ and BMI^-0.5^ for hospitalization outcome, BMI^0.5^ and BMI^0.5^*ln(BMI) for ICU admission outcome, BMI^2^ and BMI^2^*ln(BMI) for IMV outcome, and BMI^-0.5^ and ln(BMI) for death outcome.

A sensitivity analysis showed weaker associations between BMI category and severe COVID-19–associated illness when adjusted for other underlying medical conditions, particularly among patients aged ≥65 years (Supplementary Figure 1, https://stacks.cdc.gov/view/cdc/103732). Results of a second sensitivity analysis using multiple imputation for missing BMIs were consistent with the primary results (Supplementary Table and Supplementary Figure 2, https://stacks.cdc.gov/view/cdc/103732).

## Discussion

One half (50.8%) of adult COVID-19 patients in this analysis had obesity, compared with 43.1% in the total PHD-SR sample and 42.4% nationally (*5*), suggesting that adults with COVID-19–associated illness and obesity might commonly receive acute care in EDs or hospitals. The findings in this report are similar to those from previous studies that indicate an increased risk for severe COVID-19–associated illness among persons with excess weight and provide additional information about a dose-response relationship between higher BMI and risk for hospitalization, ICU admission, invasive mechanical ventilation, and death (*1*,*2*). The finding that risk for severe COVID-19–associated illness increases with higher BMI suggests that progressively intensive management of COVID-19 might be needed for patients with more severe obesity. This finding also supports the hypothesis that inflammation from excess adiposity might be a factor in the severity of COVID-19–associated illness (*3*,*8*). The positive association found between underweight and hospitalization risk could be explained by uncaptured underlying medical conditions or impairments in essential nutrient availability and immune response (*9*).

Consistent with previous studies, the dose-response relationship between risk for hospitalization or death and higher BMI was particularly pronounced among patients aged <65 years (*1*,*2*). However, in contrast to previous studies that demonstrated little or no association between obesity and COVID-19 severity among older patients (*1*,*2*), the results in this report indicate that overweight and obesity are risk factors for invasive mechanical ventilation and that obesity or severe obesity are risk factors for hospitalization, ICU admission, and death among patients aged ≥65 years. A sensitivity analysis adjusting for other underlying medical conditions found weaker associations between BMI and severe COVID-19–associated illness, which might be partially attributable to indirect effects of obesity on COVID-19 or overadjustment by including intermediate variables on the causal pathway from exposure (i.e., BMI) to outcome.

BMI is continuous in nature, and the analyses in this report describe a J-shaped association between BMI and severe COVID-19, with the lowest risk at BMIs near the threshold between healthy weight and overweight in most instances. Risk for invasive mechanical ventilation increased over the full range of BMIs, possibly because of impaired lung function associated with higher BMI (*4*). These results highlight the need to promote and support a healthy BMI, which might be especially important for populations disproportionately affected by obesity, particularly Hispanic or Latino and non-Hispanic Black adults and persons from low-income households, which are populations who have a higher prevalence of obesity and are more likely to have worse outcomes from COVID-19 compared with other populations.^¶¶¶^

The findings in this study are subject to at least five limitations. First, risk estimates for severe COVID-19–associated illness (including hospitalization) were measured only among adults who received care at a hospital; therefore, these estimates might differ from the risk among all adults with COVID-19. Second, hospitalization risk estimates might have been affected by bias introduced by hospital admission factors other than COVID-19 severity, such as a health care professional’s anticipation of future severity. Third, only patients with reported height and weight information were included; among 238 hospitals, 28% of patients were missing height information, weight information, or both. However, results of a sensitivity analysis using multiple imputation for missing BMIs were consistent with the primary findings. Fourth, the BMI of some older adults might have been misclassified because of complex interactions between height loss and sarcopenia, a condition characterized by loss of skeletal muscle mass and function (*10*). Finally, although this analysis includes one of the largest samples of patients with available heights and weights to be assessed to date, the results are not representative of the entire U.S. patient population.

The findings in this report highlight a dose-response relationship between higher BMI and severe COVID-19–associated illness and underscore the need for progressively intensive illness management as obesity severity increases. Continued strategies are needed to ensure community access to nutrition and physical activity opportunities that promote and support a healthy BMI. Preventing COVID-19 in adults with higher BMIs and their close contacts remains important and includes multifaceted protection measures such as masking, as well as continued vaccine prioritization (*6*) and outreach for this population.

**Summary**

**What is already known about this topic?**

Obesity increases the risk for severe COVID-19–associated illness.

**What is added by this report?**

Among 148,494 U.S. adults with COVID-19, a nonlinear relationship was found between body mass index (BMI) and COVID-19 severity, with lowest risks at BMIs near the threshold between healthy weight and overweight in most instances, then increasing with higher BMI. Overweight and obesity were risk factors for invasive mechanical ventilation. Obesity was a risk factor for hospitalization and death, particularly among adults aged <65 years.

**What are the implications for public health practice?**

These findings highlight clinical and public health implications of higher BMIs, including the need for intensive management of COVID-19–associated illness, continued vaccine prioritization and masking, and policies to support healthy behaviors.

## Footnotes

* Obesity (body mass index ≥30 kg/m^2^) is frequently categorized into three categories: class 1 (30.0–34.9 kg/m^2^), class 2 (35.0–39.9 kg/m^2^), and class 3 (≥40 kg/m^2^). Class 3 obesity is sometimes referred to as “extreme” or “severe” obesity.

†
https://www.cdc.gov/obesity/adult/causes.html

§ Data in PHD-SR, formerly known as the PHD COVID-19 Database, are released every 2 weeks; release date March 2, 2021, access date March 3, 2021. http://offers.premierinc.com/rs/381-NBB-525/images/PHD_COVID-19_White_Paper.pdf

¶
https://www.cdc.gov/nchs/data/icd/Announcement-New-ICD-code-for-coronavirus-3-18-2020.pdf

** Heights and weights were excluded if they were substantially larger or smaller than expected (defined as height <44 inches [112 cm] or >90 inches [229 cm]; weight <25 kg [55 lbs] or >454 kg [1,000 lbs]; and BMI <12 kg/m^2^ or >110 kg/m^2^).

†† Patients who were hospitalized were defined as those with a reported hospital inpatient encounter, patients who were admitted to an ICU or who received invasive mechanical ventilation were determined by patient billing records, and patients who died were determined by patient discharge records indicating that death that occurred in the hospital or in hospice care.

§§ Each model included the following covariates: BMI (modeled as fractional polynomials), age category, sex, race/ethnicity, payer type, hospital urbanicity, hospital U.S. Census region, and admission month. The best fitting second degree fractional polynomials of BMI were BMI^-2^ and BMI^-0.5^ for hospitalization outcome, BMI^0.5^ and BMI^0.5^*ln(BMI) for ICU admission outcome, BMI^2^ and BMI^2^*ln(BMI) for invasive mechanical ventilation outcome, and BMI^-0.5^ and ln(BMI) for death outcome. Risk was obtained as predictive margins (probability of the outcome) over the BMI range from 15 kg/m^2^ to 60 kg/m^2^. Models were then reestimated by including the interaction of BMI (as fractional polynomials) and age category (18–39, 40–49, 50–64, 65–74, and ≥75 years). Risk was estimated as predictive margins (probability of the outcome) over the BMI range from 15 kg/m^2^ to 60 kg/m^2^ and at each age category.

¶¶ Age category (18–39, 40–49, 50–64, 65–74, and ≥75 years) was included in all models except those stratified by age (<65 and ≥65 years). Cubic polynomial of age (linear, squared, and cubed terms) was included in models stratified for patients aged <65 years and ≥65 years to account for possible nonlinear associations between age and COVID-19–associated illness.

***
https://www.cdc.gov/coronavirus/2019-ncov/need-extra-precautions/evidence-table.html

††† Underlying medical conditions were defined by 1) using the ICD-10-CM Chronic Condition Indicator to identify chronic ICD-10-CM codes from January 2019 until (and including) the patient’s first health care encounter with a COVID-19 diagnosis and 2) aggregating the chronic ICD-10-CM codes into the following smaller number of meaningful categories using Clinical Classifications Software Refined (CCSR for ICD-10-CM; Agency for Healthcare Research and Quality): hypertension, CIR007 and CIR008; coronary atherosclerosis and other heart disease, CIR011; chronic kidney disease, GEN003; diabetes, END002 and END003; cancers, all CCSR categories starting with “NEO”; and chronic obstructive pulmonary disease and bronchiectasis, RSP008. ICD-10-CM codes marked as nonchronic by the Chronic Condition Indicator were excluded from the CCSR categories.

§§§ 45 C.F.R. part 46, 21 C.F.R. part 56; 42 U.S.C. Sect. 241(d); 5 U.S.C. Sect. 552a; 44 U.S.C. Sect. Sect. 3501 et seq.

¶¶¶
https://www.cdc.gov/obesity/data/obesity-and-covid-19.html
